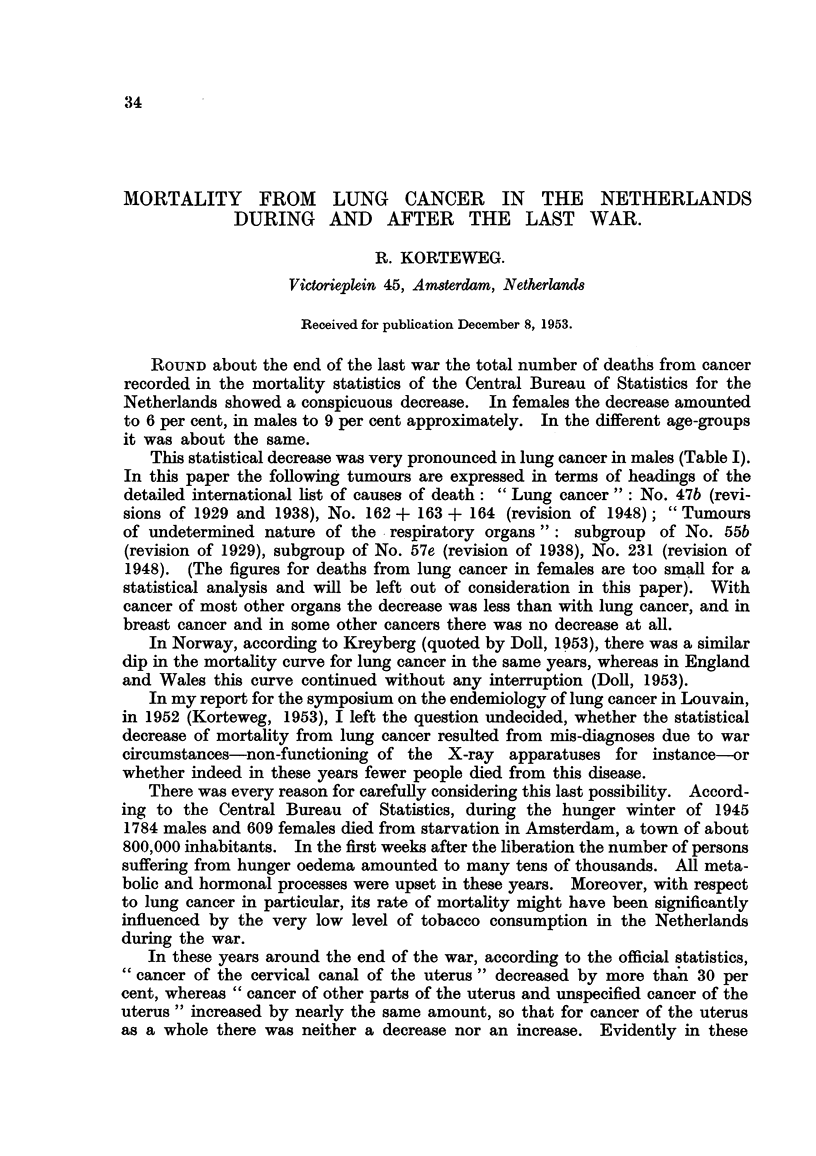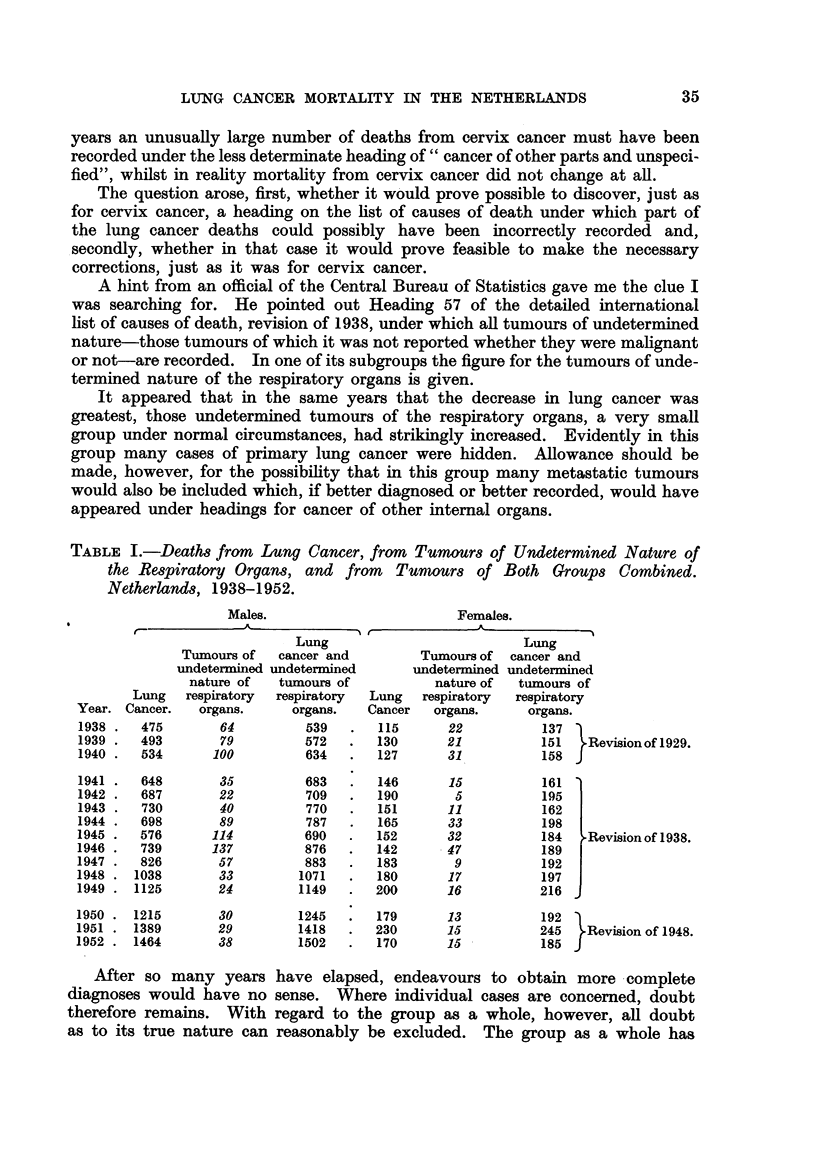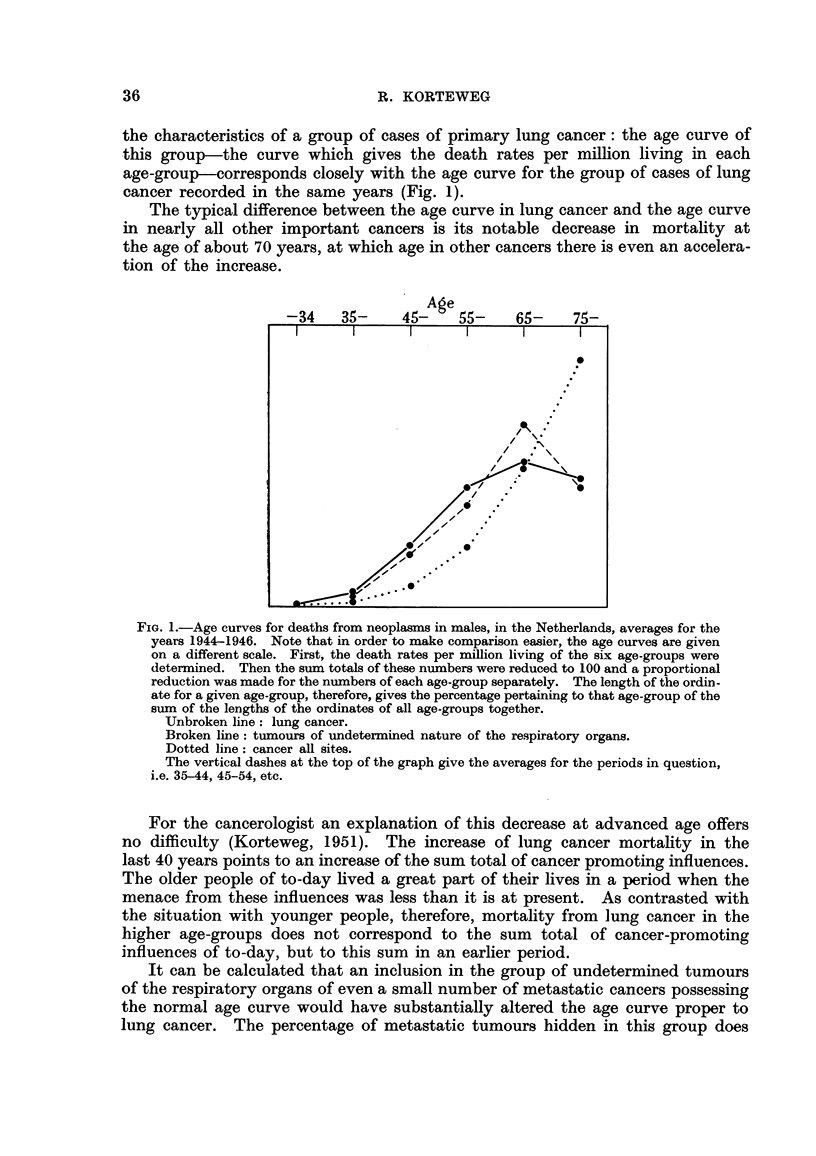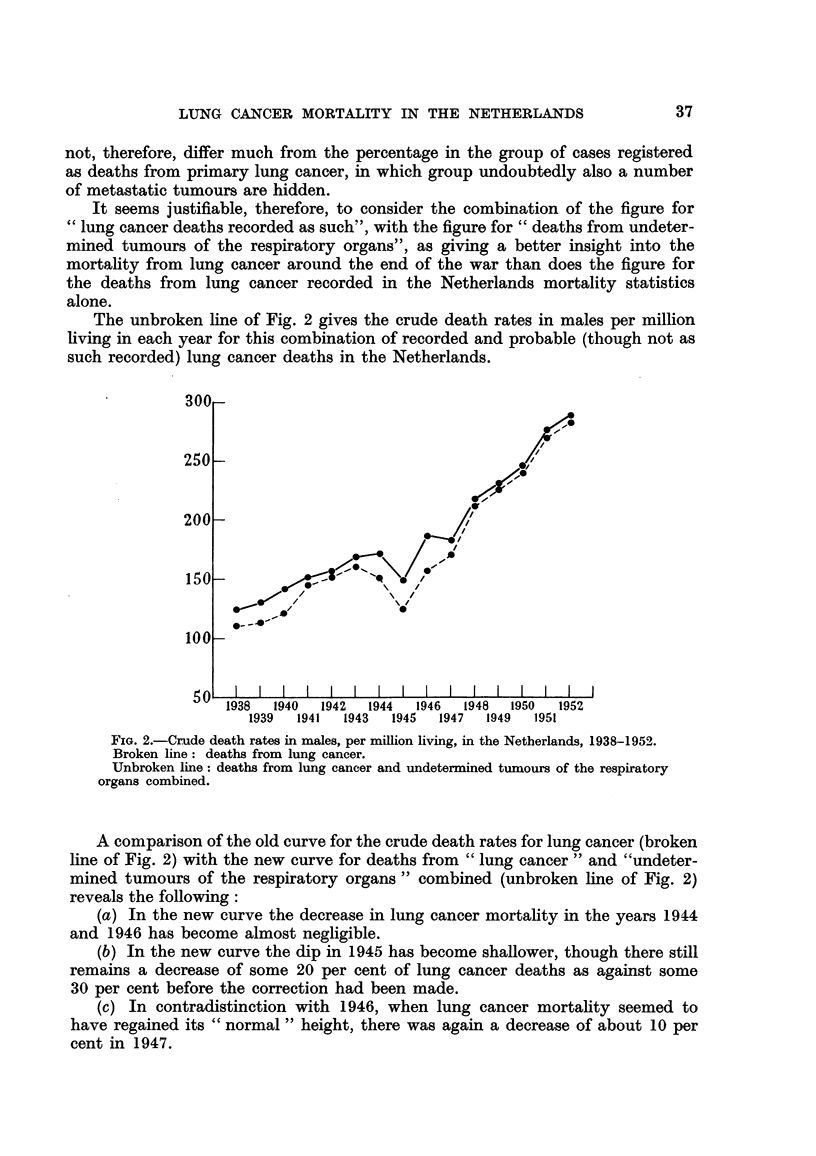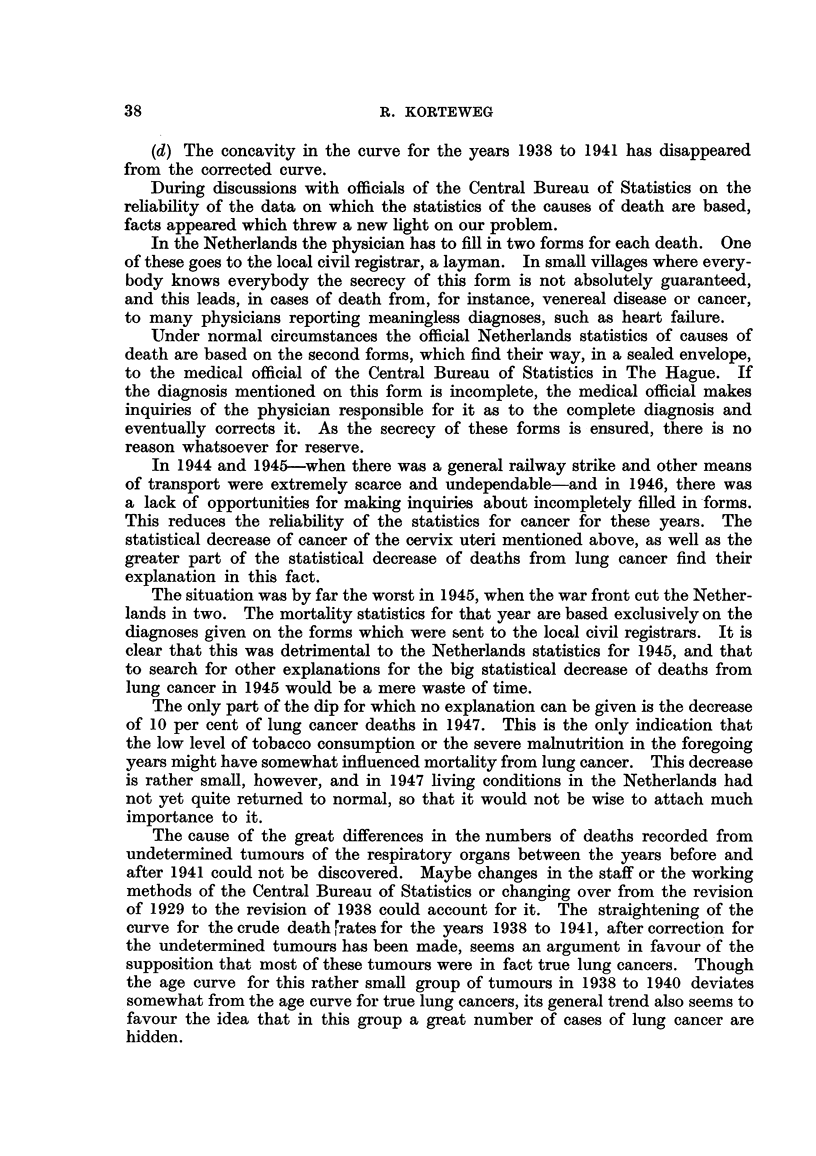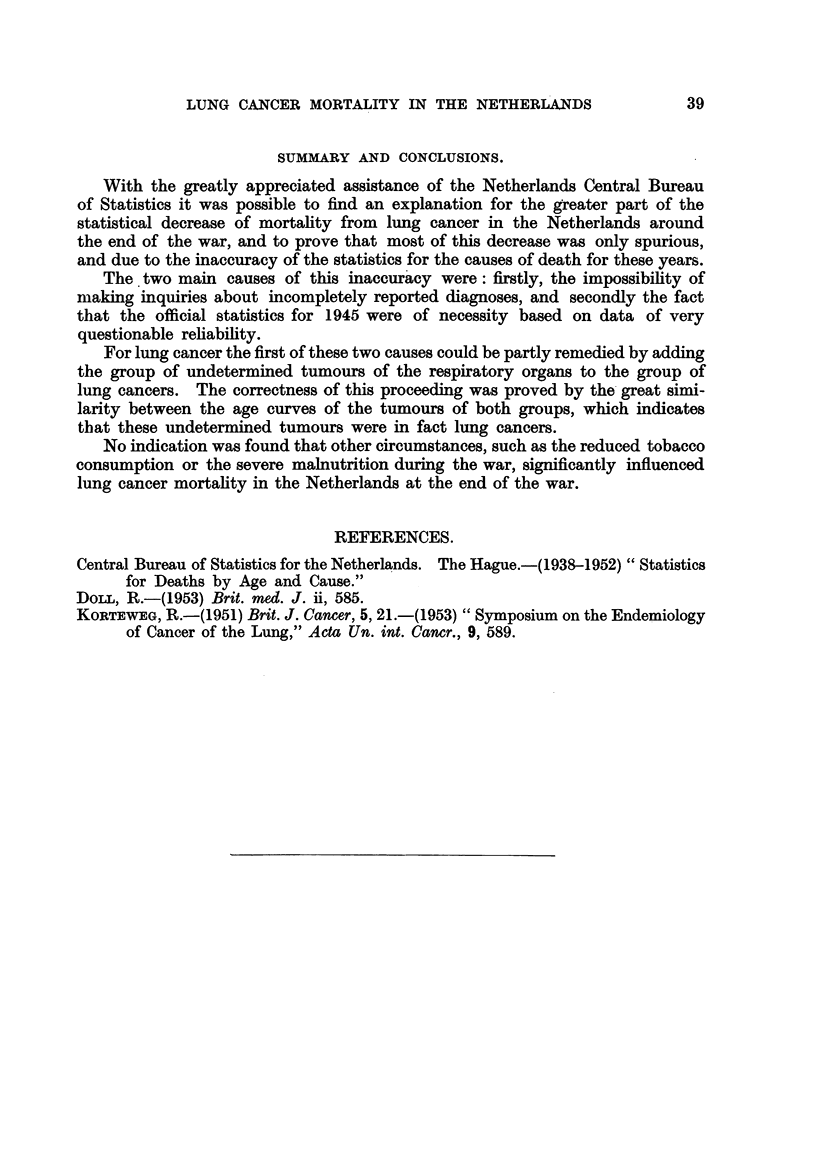# Mortality from Lung Cancer in the Netherlands during and after the Last War

**DOI:** 10.1038/bjc.1954.3

**Published:** 1954-03

**Authors:** R. Korteweg


### MORTALITY FROM LUNG CANCER IN THE NETHERLANDS DURING AND AFTER THE LAST WAR.

R. KORTEWEG.

*Victorieplein 45, Amsterdam, Netherlands*

Received for publication December 8, 1953.

ROUND about the end of the last war the total number of deaths from cancer recorded in the mortality statistics of the Central Bureau of Statistics for the Netherlands showed a conspicuous decrease. In females the decrease amounted to 6 per cent, in males to 9 per cent approximately. In the different age-groups it was about the same.

This statistical decrease was very pronounced in lung cancer in males (Table I). In this paper the following tumours are expressed in terms of headings of the detailed international list of causes of death : " Lung cancer " : No. 47*b* (revisions of 1929 and 1938), No. 162 + 163 + 164 (revision of 1948) ; " Tumours of undetermined nature of the respiratory organs " : subgroup of No. 55*b* (revision of 1929), subgroup of No. 57*e* (revision of 1938), No. 231 (revision of 1948). (The figures for deaths from lung cancer in females are too small for a statistical analysis and will be left out of consideration in this paper). With cancer of most other organs the decrease was less than with lung cancer, and in breast cancer and in some other cancers there was no decrease at all.

In Norway, according to Kreyberg (quoted by Doll, 1953), there was a similar dip in the mortality curve for lung cancer in the same years, whereas in England and Wales this curve continued without any interruption (Doll, 1953).

In my report for the symposium on the endemiology of lung cancer in Louvain, in 1952 (Korteweg, 1953), I left the question undecided, whether the statistical decrease of mortality from lung cancer resulted from mis-diagnoses due to war circumstances—non-functioning of the X-ray apparatuses for instance—or whether indeed in these years fewer people died from this disease.

There was every reason for carefully considering this last possibility. According to the Central Bureau of Statistics, during the hunger winter of 1945 1784 males and 609 females died from starvation in Amsterdam, a town of about 800,000 inhabitants. In the first weeks after the liberation the number of persons suffering from hunger oedema amounted to many tens of thousands. All metabolic and hormonal processes were upset in these years. Moreover, with respect to lung cancer in particular, its rate of mortality might have been significantly influenced by the very low level of tobacco consumption in the Netherlands during the war.

In these years around the end of the war, according to the official statistics, " cancer of the cervical canal of the uterus " decreased by more than 30 per cent, whereas " cancer of other parts of the uterus and unspecified cancer of the uterus " increased by nearly the same amount, so that for cancer of the uterus as a whole there was neither a decrease nor an increase. Evidently in these years an unusually large number of deaths from cervix cancer must have been recorded under the less determinate heading of " cancer of other parts and unspecified", whilst in reality mortality from cervix cancer did not change at all.

The question arose, first, whether it would prove possible to discover, just as for cervix cancer, a heading on the list of causes of death under which part of the lung cancer deaths could possibly have been incorrectly recorded and, secondly, whether in that case it would prove feasible to make the necessary corrections, just as it was for cervix cancer.

A hint from an official of the Central Bureau of Statistics gave me the clue I was searching for. He pointed out Heading 57 of the detailed international list of causes of death, revision of 1938, under which all tumours of undetermined nature—those tumours of which it was not reported whether they were malignant or not—are recorded. In one of its subgroups the figure for the tumours of undetermined nature of the respiratory organs is given.

It appeared that in the same years that the decrease in lung cancer was greatest, those undetermined tumours of the respiratory organs, a very small group under normal circumstances, had strikingly increased. Evidently in this group many cases of primary lung cancer were hidden. Allowance should be made, however, for the possibility that in this group many metastatic tumours would also be included which, if better diagnosed or better recorded, would have appeared under headings for cancer of other internal organs.

TABLE I.—*Deaths from Lung Cancer, from Tumours of Undetermined Nature of the Respiratory Organs, and from Tumours of Both Groups Combined. Netherlands,* 1938–1952.

| | Males. | | | Females. | | | |
|---|---|---|---|---|---|---|---|
| Year. | Lung Cancer. | Tumours of undetermined nature of respiratory organs. | Lung cancer and undetermined tumours of respiratory organs. | Lung Cancer | Tumours of undetermined nature of respiratory organs. | Lung cancer and undetermined tumours of respiratory organs. | |
| 1938 | 475 | *64* | 539 | 115 | *22* | 137 | Revision of 1929. |
| 1939 | 493 | *79* | 572 | 130 | *21* | 151 | |
| 1940 | 534 | *100* | 634 | 127 | *31* | 158 | |
| 1941 | 648 | *35* | 683 | 146 | *15* | 161 | Revision of 1938. |
| 1942 | 687 | *22* | 709 | 190 | *5* | 195 | |
| 1943 | 730 | *40* | 770 | 151 | *11* | 162 | |
| 1944 | 698 | *89* | 787 | 165 | *33* | 198 | |
| 1945 | 576 | *114* | 690 | 152 | *32* | 184 | |
| 1946 | 739 | *137* | 876 | 142 | *47* | 189 | |
| 1947 | 826 | *57* | 883 | 183 | *9* | 192 | |
| 1948 | 1038 | *33* | 1071 | 180 | *17* | 197 | |
| 1949 | 1125 | *24* | 1149 | 200 | *16* | 216 | |
| 1950 | 1215 | *30* | 1245 | 179 | *13* | 192 | Revision of 1948. |
| 1951 | 1389 | *29* | 1418 | 230 | *15* | 245 | |
| 1952 | 1464 | *38* | 1502 | 170 | *15* | 185 | |

After so many years have elapsed, endeavours to obtain more complete diagnoses would have no sense. Where individual cases are concerned, doubt therefore remains. With regard to the group as a whole, however, all doubt as to its true nature can reasonably be excluded. The group as a whole has the characteristics of a group of cases of primary lung cancer: the age curve of this group—the curve which gives the death rates per million living in each age-group—corresponds closely with the age curve for the group of cases of lung cancer recorded in the same years (Fig. 1).

The typical difference between the age curve in lung cancer and the age curve in nearly all other important cancers is its notable decrease in mortality at the age of about 70 years, at which age in other cancers there is even an acceleration of the increase.

FIG. 1.—Age curves for deaths from neoplasms in males, in the Netherlands, averages for the years 1944–1946. Note that in order to make comparison easier, the age curves are given on a different scale. First, the death rates per million living of the six age-groups were determined. Then the sum totals of these numbers were reduced to 100 and a proportional reduction was made for the numbers of each age-group separately. The length of the ordinate for a given age-group, therefore, gives the percentage pertaining to that age-group of the sum of the lengths of the ordinates of all age-groups together.

Unbroken line: lung cancer.

Broken line: tumours of undetermined nature of the respiratory organs.

Dotted line: cancer all sites.

The vertical dashes at the top of the graph give the averages for the periods in question, i.e. 35–44, 45–54, etc.

For the cancerologist an explanation of this decrease at advanced age offers no difficulty (Korteweg, 1951). The increase of lung cancer mortality in the last 40 years points to an increase of the sum total of cancer promoting influences. The older people of to-day lived a great part of their lives in a period when the menace from these influences was less than it is at present. As contrasted with the situation with younger people, therefore, mortality from lung cancer in the higher age-groups does not correspond to the sum total of cancer-promoting influences of to-day, but to this sum in an earlier period.

It can be calculated that an inclusion in the group of undetermined tumours of the respiratory organs of even a small number of metastatic cancers possessing the normal age curve would have substantially altered the age curve proper to lung cancer. The percentage of metastatic tumours hidden in this group does not, therefore, differ much from the percentage in the group of cases registered as deaths from primary lung cancer, in which group undoubtedly also a number of metastatic tumours are hidden.

It seems justifiable, therefore, to consider the combination of the figure for " lung cancer deaths recorded as such", with the figure for " deaths from undetermined tumours of the respiratory organs", as giving a better insight into the mortality from lung cancer around the end of the war than does the figure for the deaths from lung cancer recorded in the Netherlands mortality statistics alone.

The unbroken line of Fig. 2 gives the crude death rates in males per million living in each year for this combination of recorded and probable (though not as such recorded) lung cancer deaths in the Netherlands.

FIG. 2.—Crude death rates in males, per million living, in the Netherlands, 1938–1952.
Broken line : deaths from lung cancer.
Unbroken line : deaths from lung cancer and undetermined tumours of the respiratory organs combined.

A comparison of the old curve for the crude death rates for lung cancer (broken line of Fig. 2) with the new curve for deaths from " lung cancer " and "undetermined tumours of the respiratory organs " combined (unbroken line of Fig. 2) reveals the following :

(*a*) In the new curve the decrease in lung cancer mortality in the years 1944 and 1946 has become almost negligible.

(*b*) In the new curve the dip in 1945 has become shallower, though there still remains a decrease of some 20 per cent of lung cancer deaths as against some 30 per cent before the correction had been made.

(*c*) In contradistinction with 1946, when lung cancer mortality seemed to have regained its " normal " height, there was again a decrease of about 10 per cent in 1947.

(*d*) The concavity in the curve for the years 1938 to 1941 has disappeared from the corrected curve.

During discussions with officials of the Central Bureau of Statistics on the reliability of the data on which the statistics of the causes of death are based, facts appeared which threw a new light on our problem.

In the Netherlands the physician has to fill in two forms for each death. One of these goes to the local civil registrar, a layman. In small villages where everybody knows everybody the secrecy of this form is not absolutely guaranteed, and this leads, in cases of death from, for instance, venereal disease or cancer, to many physicians reporting meaningless diagnoses, such as heart failure.

Under normal circumstances the official Netherlands statistics of causes of death are based on the second forms, which find their way, in a sealed envelope, to the medical official of the Central Bureau of Statistics in The Hague. If the diagnosis mentioned on this form is incomplete, the medical official makes inquiries of the physician responsible for it as to the complete diagnosis and eventually corrects it. As the secrecy of these forms is ensured, there is no reason whatsoever for reserve.

In 1944 and 1945—when there was a general railway strike and other means of transport were extremely scarce and undependable—and in 1946, there was a lack of opportunities for making inquiries about incompletely filled in forms. This reduces the reliability of the statistics for cancer for these years. The statistical decrease of cancer of the cervix uteri mentioned above, as well as the greater part of the statistical decrease of deaths from lung cancer find their explanation in this fact.

The situation was by far the worst in 1945, when the war front cut the Netherlands in two. The mortality statistics for that year are based exclusively on the diagnoses given on the forms which were sent to the local civil registrars. It is clear that this was detrimental to the Netherlands statistics for 1945, and that to search for other explanations for the big statistical decrease of deaths from lung cancer in 1945 would be a mere waste of time.

The only part of the dip for which no explanation can be given is the decrease of 10 per cent of lung cancer deaths in 1947. This is the only indication that the low level of tobacco consumption or the severe malnutrition in the foregoing years might have somewhat influenced mortality from lung cancer. This decrease is rather small, however, and in 1947 living conditions in the Netherlands had not yet quite returned to normal, so that it would not be wise to attach much importance to it.

The cause of the great differences in the numbers of deaths recorded from undetermined tumours of the respiratory organs between the years before and after 1941 could not be discovered. Maybe changes in the staff or the working methods of the Central Bureau of Statistics or changing over from the revision of 1929 to the revision of 1938 could account for it. The straightening of the curve for the crude death rates for the years 1938 to 1941, after correction for the undetermined tumours has been made, seems an argument in favour of the supposition that most of these tumours were in fact true lung cancers. Though the age curve for this rather small group of tumours in 1938 to 1940 deviates somewhat from the age curve for true lung cancers, its general trend also seems to favour the idea that in this group a great number of cases of lung cancer are hidden.

### SUMMARY AND CONCLUSIONS.

With the greatly appreciated assistance of the Netherlands Central Bureau of Statistics it was possible to find an explanation for the greater part of the statistical decrease of mortality from lung cancer in the Netherlands around the end of the war, and to prove that most of this decrease was only spurious, and due to the inaccuracy of the statistics for the causes of death for these years.

The two main causes of this inaccuracy were : firstly, the impossibility of making inquiries about incompletely reported diagnoses, and secondly the fact that the official statistics for 1945 were of necessity based on data of very questionable reliability.

For lung cancer the first of these two causes could be partly remedied by adding the group of undetermined tumours of the respiratory organs to the group of lung cancers. The correctness of this proceeding was proved by the great similarity between the age curves of the tumours of both groups, which indicates that these undetermined tumours were in fact lung cancers.

No indication was found that other circumstances, such as the reduced tobacco consumption or the severe malnutrition during the war, significantly influenced lung cancer mortality in the Netherlands at the end of the war.

### REFERENCES.

Central Bureau of Statistics for the Netherlands. The Hague.—(1938–1952) " Statistics for Deaths by Age and Cause."

Doll, R.—(1953) *Brit. med. J.* ii, 585.

Korteweg, R.—(1951) *Brit. J. Cancer*, **5**, 21.—(1953) " Symposium on the Endemiology of Cancer of the Lung," *Acta Un. int. Cancr.*, **9**, 589.